# Insect repellent plants traditional usage practices in the Ethiopian malaria epidemic-prone setting: an ethnobotanical survey

**DOI:** 10.1186/1746-4269-10-22

**Published:** 2014-02-12

**Authors:** Kaliyaperumal Karunamoorthi, Teklu Hailu

**Affiliations:** 1 Unit of Medical Entomology and Vector Control, Department of Environmental Health Sciences and Technology, College of Public Health & Medical Sciences, Jimma University, Jimma, Ethiopia

**Keywords:** Plant-based insect repellent, Ethnobotanical survey, Knowledge, Self-reported practice

## Abstract

**Background:**

The usage of insect repellent plants (IRPs) is one of the centuries-old practices in Africa. In Ethiopia, malaria remains a leading cause of morbidity and mortality, subsequently the majority of people have a tendency to apply various plants as repellents to reduce or interrupt the biting activity of insects. Accordingly, this survey was undertaken to document and evaluate knowledge and usage practices of the local inhabitants on IRPs in the malaria epidemic-prone setting of Ethiopia.

**Methods:**

Ethnobotanical survey was conducted between January and May 2013. Selected 309 household members were interviewed by administering pre-tested questionnaire on knowledge and usage practices of repellent plants, in Bechobore Kebele, Jimma Zone, Ethiopia.

**Results:**

Overall, 70.2% (217/309) and 91.8% (199/217) of the respondents have had ample awareness and usage practices of repellent plants, respectively. Informants cited about twenty-two plant species as repellents and also indicated that these plants are useful(85.5%), accessible(86.8%), and affordable(83.9%) too. Residents mainly applying dried leaves [93.9% (187/199)] by means of burning/smouldering [98.9% (197/199)] with the traditional charcoal stove to repel insects, primarily mosquitoes. About 52.8% (105/199) of the informants using aproximately15g of dried plant-materials every day. A Chi-square analysis shows statistically a significant link between the knowledge on repellent plants and gender as well as average monthly income although not with the age of the respondents. Nevertheless, the repellent plant usage custom was not significantly associated with gender, monthly income, and age of the informants.

**Conclusion:**

Though most of the people have had an adequate awareness still a sizable faction of society suffers with deprivation of IRPs knowledge and usage practices. Therefore, this study calls for more surveys to conserve the existing indigenous knowledge and cultural practices. It could lay the first stone to develop the next generation cost-effective vector control tools in the near future.

## Background

Insect-transmitted diseases cause over a million deaths and threaten hundreds of millions lives every year [1]. In the recent decades, the global warming, unplanned urbanization, and unchecked anthropogenic activities has contributed to the emergence and resurgence of many insect-borne diseases like malaria, filariasis, dengue fever, leishmaniasis, trypanosomiasis, and several Arbo-viral diseases [2]. Malaria is a life-threatening disease [3], and the malaria parasites are transmitted via mosquitoes. It remains as one of the leading public health issues in resource-poor settings of sub-Saharan Africa (SSA), Southeast Asian (SEA) countries and beyond [4]. The recent World Malaria Report [1] has estimated that in 2010 globally around half of the world population (approximately 3.3 billion people) has been at the risk of infection in 104 countries, and about 219 million clinical cases have been reported, It contributes to approximately 660,000 deaths worldwide among which 91% of deaths occur in Africa alone.

It has been estimated that approximately 20-30% of all African malaria cases occur in Nigeria and Ethiopia alone [5]. Ethiopia is among the most malaria epidemic-prone countries in sub-Saharan region. During the epidemic, the rates of morbidity and mortality are observed to raise dramatically (i.e. 3–5 fold) [6]. Nearly 52 million of people (68%) live in malarious areas [6]. It remains as a major cause of maternal and childhood morbidity and mortality [7] due to lesser immunity among the expectant mother and children than others [8]. It is a major impediment to socioeconomic development as the peak transmission season coincides with the usual planting and harvesting periods [9].

In Ethiopia, *Anopheles arabiensis* Patton is a predominant malaria vector, while *An. funestus* Giles, *An. pharoensis* Theobald, and *An. nili* Theobald are the secondary vectors [10]. *An. arabiensis* has the ability, to adapt to all types of climatic features and in order to avoid insecticide treated surfaces, it can quickly adjust from endophagic to exophagic nature too. It is important to emphasize that though *An. arabiensis* biting occurs all through night, the peak man-biting activity begins in the early evening (19:00). It ultimately circumvents some of the protective effects of bed nets [11] and other personal protection interventions. In this context, repellent has a pivotal role to play on ensuring to minimize the insects hazard and disease transmission [12].

The repellent plant usage is intertwined with Africa’s tradition and culture [13] for instance, in Eretria people just hang them around the bed, doors and windows [14], in Ethiopia and Kenya people burn or spray a number of plants to reduce the numbers of mosquitoes indoors at night [13,15-18]. In Ethiopia, burning of dried repellent plants is one of the common phenomena to drive away insects and mosquitoes. It is usually performed by using the traditional charcoal stove (thermal expulsion) in the early evenings. In the recent years, a revived interest has been observed among the health-conscious consumers with the plant-based repellents because of their low mammalian and non-target toxicity [17] than their synthetic counterparts. Consequently, the exploding demands and falling supply insists to conduct more ethnobotanical survey in order to formulate risks-reduced/green pesticides and repellents from the traditionally used repellent plants.

The interaction between people and plants is called ethnobotany [19] and it is a tool to unlock the secrecy of indigenous knowledge and cultural practices for the well-being of mankind. Repellent plant usage custom has been developed, sustained and passed down to many generations within a community mostly through word of mouth [17]. The practical knowledge and practices often adapt or get modified according to the current needs too. Subsequently, these may contribute to distortion or gradual erosion of indigenous knowledge and cultural practices and therefore it has to be tapped properly. From these perspectives, the purpose of the present survey was to assess the knowledge and usage practices of repellent plants among the local inhabitants in a malaria-epidemic prone area of Ethiopia. This spinoff research work could open the door to pick and choose a repellent plant among the countless promising candidates and to explore and develop long lasting and cost-effective vector control tools in the future.

## Materials and methods

### Description of the ethnobotanical survey setting

**Figure 1:**
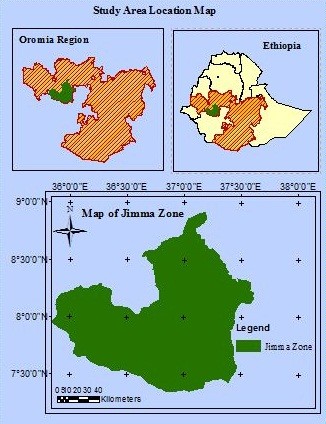
Location of the study area Bechobore kebele, Jimma Zone, Oromia region, Ethiopia.

The ethnobotanical survey was conducted among the Bechobore kebele [village; (small local administrative unit in Ethiopia)], residents. It is one of the malarious areas in Jimma Zone, Kersa woreda (district), Oromia Regional State of Southwestern Ethiopia (Figure 1). It is located 356 km away from Addis Ababa, the federal capital of Ethiopia. The Oromo ethnic group is the most predominant one (81.2%) and the majority of them are Muslims [20]. It is located at an altitude of 1755 m above sea level and the average annual rainfall and temperature is about 700 mm and 21°C, respectively. Based on climatic conditions, the study area classified as one of the woienadega areas of Ethiopia, where malaria is holo and hyper-endemic (intense transmission) in nature. One health post and one middle school are located. The kebele is divided into seven zones for administrative purpose. Among them, three (i.e. Saxama, Sedecha and Tulama) zones are highly malarious where disease transmission occurs almost throughout the year.

Malaria is a major public health issue with more than 56% of clinical case incidences at household level (personal communication with the local Health Extension Worker (HEW) in April 2013). The local residents cultivate Teff (*Eragrostis tef* Zucc.), maize (*Zea mays* L.), barley (*Hordeum vulgare* L.), cereal such as sorghum (*Sorghum bicolor* L.), wheat (*Triticum aestivum* L.), etc. Besides, it is also renowned for the cultivation of the cash crop like Khat (*Catha edulis* Forsk.) and coffee (*Coffea arabica* L.) and raising live stocks. Boye river/wetland is running perennial along the entire study area and during the rainy season it creates a swampy or marshy area and more or less permanent large pools of water or small ‘lakes’, which serve as potential breeding sites for mosquitoes [21]. The greater majority of the houses are called traditional tukuls, built with mud and covered with a thatch roofs.

Due to the prolonged period of exposure to malaria, residents have traditionally been applying several plants as repellents to drive away blood-sucking insects, particularly mosquitoes. This practice is quite interlinked with the centuries-old Ethiopian coffee ceremony. The present study setting (an Ethiopian region called, Kaffa) being the birthplace of the coffee plant, this ceremony is practiced by making coffee from a traditional charcoal stove, which is continuing to burn the traditional incenses. It lasts almost 40 min to 2 h by spreading of fresh aromatic grasses and/or flowers across the floor. In most parts of Ethiopia, the observance takes place three times a day - in the morning, at noon and evening. Therefore, we strongly believe that the repellent usage practices could have emerged from the Ethiopian coffee ceremony ritual.

### Study design and sampling technique

The study was a community based cross-sectional survey conducted between January and May of 2013. A stratified, systematic random sampling was used for the selection of a total of 309 households from three (i.e. Saxama, Sedecha and Tulama) zones of the Bechobore Kebele (village). 103 respondents were chosen from each of the selected zones. The sample size was calculated by employing 95% confidence interval formula to estimate a population proportion.

### Interview

The interview was carried out by involving selected 309 household members. In order to evaluate the clarity of the questionnaire, the validity of the instrument, and reactions of the respondents to the questionnaire a pre-test was conducted prior to the actual data collection on 10% of the study population, i.e. about 31 residents by the enumerators, in an area different from the study area, but with the similar socio-demographic pattern. One adult from each selected household was interviewed on the knowledge and traditional uses of repellent plants, by administering a pre-tested questionnaire specifically designed for this purpose. Male and female respondents from all age-groups were included. To avoid biassing information and variables, the questionnaire has been prepared in the English language and has been translated into the local native language (Afan Oromo) in order to make it easy to understand and to administer by interviewers and interviewees.

### Ethics statement

The study design and the consent process have been approved by the ethical clearance committee of the Jimma University, Ethiopia. Before the commencement of the survey, meetings with community health workers, community leaders and members of the neighbourhood associations were held in which the objectives of the survey were clearly explained. Since all the selected respondents were above eighteen*-*years of age, the informed written consent was obtained from each of the study participants prior to the interview, with the help of an approved voluntary consent form. Every participant was assured to withdraw the interview at any phase if they wish to do so. However, all the informants actively participated and no one declined to cease the interview. Study identification numbers were used instead of participant names and the information collected has been kept confidential. Feedback to the study population was conducted in the form of dissemination meetings after the completion of the survey.

### Ethnobotanical data collection

A team of well-trained and closely supervised local interviewers conducted the household survey using a pre-tested questionnaire to interview with the representative of selected household. Interviewers collected information regarding socio-demographic and ethnobotanical data. Study participants were asked to impart their knowledge and usage custom on repellent plants. The main questions focused on (1) the usage and knowledge on IRPs, (2) names of plants used or known, (3) insects against which plants are used, (4) mode of applications, and (5) parts of the plant material used. Besides, the repellent plants have been categorized basis on their affordability, accessibility and efficiency for the assessment by employing the following criterion; (a) potentially useful (the plants potent enough to drive away insects’ minimum of 60 min), (b) accessible (the plants available within their neighbourhood without any serious efforts), and (c) affordable (the cost is within the range of 1–3 Ethiopian birr).

The authors also made personal observations in the field on the typical habitats and repellent plants collected by accompanying traditional users, translators and field assistants. Specimens of the reported plants were collected during the regular walk in the fields. The collected voucher specimens were pressed, numbered, dried, identified and deposited at the Jimma University Regional Herbarium and at The National Herbarium (ETH) in Addis Ababa University. Identification of specimens was done with the help of herbarium materials, experts and taxonomic keys in the Flora of Ethiopia and Eritrea [22-28].

### Data management and analysis

In the field, data were collected in a standardized questionnaire and data collection forms and checked for errors and completeness. Data was then counterchecked before entry into DbaseV (Borland International, Scotts Valley, California, USA) using the double entry system. Summary statistics were performed using STATA version 10 (STATA Corp., Texas, USA). The range and mean were analyzed and appropriate tables, graphs and percentage details were displayed. The chi-square analysis was performed to test the hypothesis. The level of significance was also determined by using 95% of confidence intervals and *P-*value.

## Results and discussion

### Socio-demographic characteristics of respondents

The socio-demographic characteristics of the study respondents are shown in the Table 1 Overall, 70.2%(217/309) of the local inhabitants have had ample awareness, nevertheless, 91.8%(199/217) of them were applying these plants as insect repellents (Table 1). It is important to note that though the survey has been conducted by involving 309 eligible respondents, only 217 of them have had awareness on insect repellent plants accordingly the level of awareness was 70.2%. However, out of 217 knowleged residents 199 of them were using (91.8) these plants as insect repellents.

**Table 1:** Study of respondents with gender, age, educational status, average monthly income, religion, ethnicity, family size and knowledge of insect repellent plants among the local inhabitants in the Becho Bore Kebele

**Socio-demographic characteristics variables**	**Frequency (n = 309)**	**Percent**
**Gender**		
Male	108	34.9
Female	201	65.1
**Age of respondents (in Years)**		
19-30	107	34.7
31-40	104	33.6
41-50	46	14.8
51-60	11	03.6
≥60	41	13.3
**Occupational status**		
Peasant (Small farmers)	50	16.2
Merchant	57	18.4
Civil servants	45	14.5
Student	11	03.6
Housewife	46	14.9
Daily labour	52	16.9
Other	48	15.5
**Educational status**		
Illiterate	67	21.7
Read write	26	08.6
Grade 1-5	37	11.9
Grade 6-8	77	24.6
Grade 9-10	52	16.8
Grade 11-12	5	01.8
College & Above	45	14.6
**Monthly income** [Ethiopian Birr (1 USD = 19.7 Eth Birr)]		
<200	25	08.1
201-400	148	47.8
401-600	42	13.5
601-800	68	22.1
>800	26	08.5
**Knowledge on insect repellent plants (n = 309)**		
Yes	217	70.2
No	92	29.8
**Usage custom of insect repellent plants (n = 217)**		
Yes	199	91.8
No	18	08.3

### Knowledge on traditional insect repellent plants

Respondents cited overall 23 plants as insect repellents to repel insects, principally mosquitoes (Table 2). Nevertheless, one of the most renowned traditional fish poisoning plants called Birbira [vernacular name (local native language, Amharic); *Milletia ferruginea*] was cited by three study participants by mistake as insect repellent. Consequently, this plant was excluded and finally all the known 22 species were compiled in the Table 2. Interestingly 21.7%( 43/199) of the respondents were using Shita (a mixture of various repellent plants stem, root, resin, leaves and bark) which is abundantly available in most of the Ethiopian towns. It is mainly prepared by the folk with approximately 5 g of repellent plant materials wrapped in a plastic paper and commercialized.

**Table 2:** Information on insect repellent plants in relation with plant parts used, method of application and types of insects repelled

**S. No.**	**Vernacular name (Afaan Oromoo)**	**Family name**	**Plant Scientific name**	**Voucher No.**	**UR (n = 199)**	**(%)**^**a**^	**Plant Part(s) used**	**Method of application**	**Insect(s) control**
1	Dhumugaa	Acanthaceae	*Justicia schimperiana* T.	JER13	57	28.7	Leaves	Burning to generate smoke.	Mosquitoes and coachroaches
2	Qullubii adii	Alliaceae	*Allium sativum* Linn.	JER17	62	31.2	Bulb	Crushing and applying the juice on the skin.	Mosquitoes
3	Eebicha	Asteraceae	*Vernonia amygdalina* Del.	JER8	71	35.7	Leaves and barks	Crushing the leaves and apply the juice on the exposed parts of the body.	Tick, mites and mosquitoes
4	Qabaaricho	Asteraceae	*Echinops kebericho* Mesfin.	JER15	60	30.2	Root	Dried parts burned to generate smoke	Mosquitoes
5	Fexo	Brassicaceae	*Lepidium sativum* Linn	JER12	51	25.7	Seeds	Crushing and applying on skin also drinking	Mosquitoes, housefly, ticks and mites.
6	Sanaficaa	Brassicaceae	*Brassica nigra* Linn. Koch	JER20	31	15.6	Seeds	Seed crushed and its juice rubbed on the body	Mosquitoes
7	Qomonyoo	Buddlejaceae	*Buddleja polystachya* Fresen.	JER19	59	29.7	Dermis of roots	Burning the dried roots to generate smoke.	Mosquitoes
8	Ixanaa( nadii)	Burseraceae	*Boswellia papyrifera* (Del.) Hochst.	JER10	98	49.3	Barks and Resin	Burning to barks and resin to generate smoke.	Mosquitoes and house fly
9	Papayaa	Caricaceae	*Carica papaya* Linn.	JER2	56	28.2	Leaves	Crushing the dried leaves and apply the juice on the exposed parts of the body.	Mosquitoes and ticks
10	Bukbuka	Colchicaceae	*Colchicum autumnale* Linn.	JER1	53	26.7	Barks/dermis	Burning the dried parts to generate smoke.	
11	Gatirra Habasha	Cupressaceae	*Cupressus lusitanica* Mill.	JER6	143	71.9	Leaves, dermis, barks	Burning dried parts to generate smoke.	Mosquitoes and house fly
12	Bakanissa	Euphorbiaceae	*Croton macrostachyus* Hochst. ex Del.	JER4	87	43.8	Leaves	Burning the dried leaves to generate smoke.	Mosquitoes
13	Qobo	Euphorbiaceae	*Ricinus communis* Linn.	JER9	54	27.2	Seeds	Seed crushed and it juices applied on the skin.	Tick, mosquitoes, and bedbugs
14	Damakessie	Lamiaceae (alt. Labiatae)	*Ocimum lamiifolium* Hochst. ex Benth.	JER3	65	32.7	Leaves	Burning dried parts to generate smoke, making juice and applying on skin	Mosquitoes
15	Qoricha michii	Lamiaceae (alt. Labiatae)	*Ocimum suave* Willd.	JER7	61	30.7	Growing plant nearby houses, whole plant and leaves	Burning dried parts to generate smoke, making juice and applying on skin	Mosquitoes
16	Hincinnii	Malvaceae	*Pavonia urens* Cav.	JER16	47	23.7	Leaves	Burning to generate smoke.	Mosquitoes and house fly
17	Akaakltii adii	Myrtaceae	*Eucalyptus globulus* Labill	JER22	112	61.4	Whole plant and leaves	Burning whole plant and crushing leaves and applying on exposed body parts	Mosquitoes and other haematophagous insects
18	Bargamoo adii	Myrtaceae	*Eucalyptus citriodora* Hook.	JER11	59	29.7	Leaves	Crushing and applying on skin and burning to generate smoke.	Mosquitoes, coachroaches, ticks and house fly
19	Ejersaa	Oleaceae	*Olea europaea* Linn.	JER18	58	29.1	Leaves and parks	Dried parts burned to generate smoke.	Mosquitoes and house fly
20	Qolaa burtukanaa	Rutaceae	*Citrus sinensis (*L.) Osb.	JER21	69	34.7	Peals	Dried peels burned to generate smoke	Mosquitoes and house fly
21	Lommii	Rutaceae	*Citrus aurantifolia* (Christm.)	JER14	24	12.1	Peels of fruits	Crushing and applying on exposed parts of the body.	Mosquitoes
22	Hargessa dhala	Xanthorrhoeaceae	*Aloe pulcherrima* M.G. Gilbert & Sebsebe.	JER5	66	33.2	Leaves	Burning the dried leaves to generate smoke and crushing leaves to spray in and around houses.	Tick and mosquitoes
23	Shita^b^	NA	NA	NA	43	21.7	Churn of several repellent plant parts	Smoking and spray	Mosquitoes and other haematophagous insects

**Note:** NA: the relevant information is not available.

UR: (use-record) the number of the respondents who claimed the use of specific plant as an insect repellent

^a^Percent does not add up to 100, because of multiple responses.

^b^Shita is a mixture of various traditional repellent plant parts such as stem, root, resin, leaves and bark. It is widely available in the marketplace in the majority of the Ethiopian towns.

### Common mosquito avoiding self-reported practices and perception

**Figure 2:**
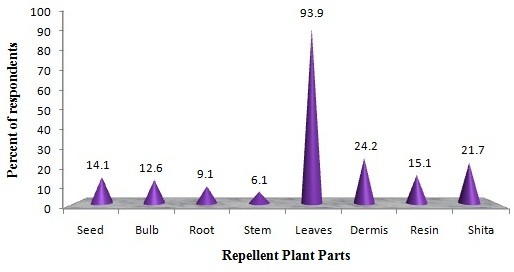
**Parts of repellent plants used by the community to drive-away different types of medically important insects and mosquitoes. Note:** Percent does not add up to 100, because of multiple responses.

**Figure 3:**
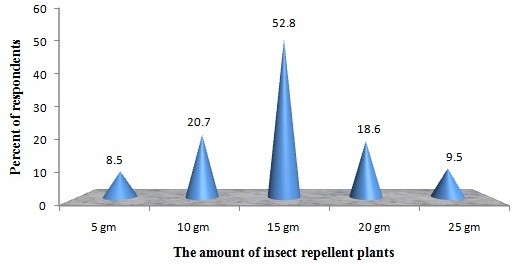
The amount of insect repellent plants used by the community repel different types of insects and mosquitoes.

The greater majority of the study participants drive-away the insects by smoldering [98.9% (197/199)] the dried leaves [93.9% (187/199)] in the early evening to minimize man-vector contact (Figure 2). Overall, 85.5%, 86.8% and 83.9% of the respondents consider that these plants are extremely useful, accessible and affordable, respectively (Table 3). About 52.8% of the local residents were applying approximately 15 g of dried plant-products every day (Figure 3). The association between respondent’s knowledge and self-reported usage practices of repellent plants with their age, gender, monthly income and educational status were tested with chi-square analysis and the results are given in Table 4.

**Table 3:** Perception of the study participants regarding the traditional insect repellent plants accessibility, affordability, effectiveness and self reported plant specieses, which are scientifically reported and authenticated as insect repellent plants

**S. No.**	**Scientific name**	**Respondents using**		**Is it potentially useful?**		**Is it accessible?**		**Is it affordable?**		**Previously reported or authenticated.**
		**UR (n = 199)**	**Percent**^**a**^	**Yes**	**No**	**Yes**	**No**	**Yes**	**No**	
1.	*Allium sativum*	62	31.2	85.4	14.6	80.6	19.4	88.7	11.3	Valerio and Maroli, [29]
2.	*Aloe pulcherrima*	66	33.2	93.9	06.1	78.7	21.3	68.1	31.9	Bekele et al., [30]
3.	*Boswellia papyrifera*	98	49.3	65.9	34.1	86.8	13.2	75.5	24.5	Karunamoorthi et al., [17]
4.	*Brassica nigra*	31	15.6	80.6	19.4	74.1	25.9	93.5	06.5	Bekele et al., [30]
5.	*Buddleja polystachya*	59	29.7	88.1	11.9	84.7	15.3	77.9	22.1	NA
6.	*Carica papaya*	56	28.2	78.6	21.4	85.8	14.2	92.8	07.2	Kazembe et al., [31]; Rawani et al., [32]
7.	*Citrus aurantifolia*	24	12.1	87.6	12.4	75.1	24.9	83.4	16.6	NA
8.	*Citrus sinensis*	69	34.7	84.1	15.9	94.2	05.8	89.9	10.1	Zewde and Jembere, [33]
9.	*Colchicum autumnale*	53	26.7	92.4	07.6	88.7	11.3	79.2	20.8	NA
10.	*Croton macrostachyus*	87	43.8	68.9	31.1	83.9	16.1	72.4	27.6	Karunamoorthi and Ilango, [3]
11.	*Cupressus lusitanica*	143	71.9	81.9	18.1	78.3	21.7	94.4	05.6	Karunamoorthi et al., [17]
12.	*Echinops kebericho*	60	30.2	88.3	11.7	95.1	4.9	90.1	09.9	Karunamoorthi et al., [15]
13.	*Eucalyptus citriodora*	59	29.7	89.9	10.1	84.7	15.3	81.3	18.7	Palsson and Jaenson, [29]
14.	*Eucalyptus globulus*	112	61.4	85.8	14.2	99.1	00.9	93.7	06.3	Kweka et al., [34]; Palsson and Jaenson, [29]
15.	*Justicia schimperiana*	57	28.7	92.9	07.1	87.8	12.2	80.8	19.2	NA
16.	*Lepidium sativum*	51	25.7	90.1	09.9	96.1	03.9	74.5	25.5	Karunamoorthi and Husen, [18]
17.	*Ocimum lamiifolium*	65	32.7	83.1	16.9	92.3	07.7	95.3	04.7	Bekele et al., [30]
18.	*Ocimum suave*	61	30.7	83.7	16.3	90.1	09.9	78.6	21.4	Kweka et al., [34]; Seyoum et al., [13];
19.	*Olea europaea*	58	29.1	84.4	15.6	89.7	10.3	87.9	12.1	Karunamoorthi et al., [15]
20.	*Pavonia urens*	47	23.7	93.7	06.3	89.3	10.7	80.9	19.1	NA
21.	*Ricinus communis*	54	27.2	88.9	11.1	92.5	07.5	83.3	16.7	Bekele et al., [30]
22.	*Vernonia amygdalina*	71	35.7	84.6	15.4	78.8	21.2	84.5	15.5	Onunkun, [35]
23.	Shita^b^	43	21.7	95.4	04.6	88.4	11.6	81.4	18.6	Karunamoorthi and Husen, [18]
**Total**				1968.2	331.8	1994.8	305.2	1928.1	371.9	
**Percent**				85.5	14.5	86.8	13.2	83.9	16.1	

**Note:** UR: (use-record) the number of the respondents who claimed the use of plant as an insect repellent

^a^Percent does not add up to 100, because of multiple responses.

^b^Shita is a mixture of various traditional repellent plant parts such as stem, root, resin, leaves and bark. It is widely available in the marketplace in the majority of the Ethiopian towns.

NA: Not available.

**Table 4:** Knowledge and usage custom of insect/mosquito repellent plants in relation with gender, age and monthly income of the respondents

**Variables**	**Number of respondents**	**Knowledge on insect repellent plants**		***P*- value**	**Insect repellent usage practices**		***P*- value**
		**Yes** (n = 217)	**No** (n = 92)		**Yes** (n = 199)	**No** (n = 18)	
**Gender**				P - 0.0209*			P - 0.4420
Female	201	150	51	χ^2^ =5.326	139	11	χ^2^ = 0.591
Male	108	67	41	*df* = 1	60	07	*df* = 1
**Age (Years)**							
18-30	107	76	31	P - 0.9979	69	07	P - 0.8799
31-40	104	72	32	χ^2^ = 0.13	67	05	χ^2^ = 1.189
41-50	46	32	14	*df* = 4	29	03	*df* = 4
51-60	11	08	03		8	00	
>60	41	29	12		26	03	
**Average monthly income** [Ethiopian Birr (1 USD = 19.7 Eth Birr]							
<200	25	18	07	P - 0.0226*	16	02	P - 0.9912
201-400	148	111	37	χ^2^ = 11.375	102	09	χ^2^ = 0.277
401-600	42	29	13	*df* = 4	27	02	*df* = 4
601-800	68	48	20		44	04	
>801	26	11	15		10	01	

**Note:** *P < 0.05 statistically significant.

This survey is intended to shed the limelight to showcase the Ethiopian people ethnobotanical knowledge and their insects avoiding practices in one of the malaria epidemic-prone settings. It is important to note that the level of awareness is relatively lower when compare with the several prior Ethiopian studies conducted in Addis Zemen town (97.2%) [16], Kofe kebele (83.6%) [17], and the Western Hararghe Zone (92.1%) [18]. It could be possibly explained that though the present survey was conducted in the rural setting, it is located in close proximity of the Jimma town. Therefore, the residents might have greater access to procure other modern personal protective tools.

Traditional Knowledge (TK) is built upon the long-term experiences and trial or error close observation by the local communities. However, over the past decades a steady decline of TK has been reported worldwide [36-38]. It is attributable to that the majority of resourceful persons often elders are not willing to impart their knowledge to others except for their eldest son or the other next of kin in order to maintain the secrecy [39]. Besides, the younger generation does not have shown up enough interest to learn/know about the value of traditional repellent/medicinal plants. Therefore, every effort must be done to protect*,* preserve, promote, and practice our TK for the betterment of mankind.

Though residents have a low-level of awareness they were using 22 repellent plants (Table 2) than the previous Ethiopian ethnobotanical surveys that have reported a maximum of just 14 plant species [16-18]. Since the study area is well-known for the diversity of various plant species, it provides an ideal opportunity for local residents to apply several plants as these are easily accessible and freely available almost throughout the year [17]. Interestingly, the majority of plants cited by the respondents have been reported and scientifically authenticated formerly by several researchers [3,13-15,29-35,40] as potent repellent and insecticidal agents against various insects, chiefly mosquitoes (Table 2). It evidently suggests that the local residents are gifted with sound knowledge and aptly applying these plants as repellents. The plant kingdom is a potential warehouse to identify several potential eco-user-friendly insect repellent/insecticides in the future. Ethiopia remains regardded as a repository of repellent plants owing to its varied climatic and topographic features [16-18].

Burning/smouldering of the dried leaves was the most common practice to prevent insects’ nuisance (Table 2 and Figure 2). The findings are quite concurrent with the numerous previous studies conducted in Ethiopia [16-18], Eritrea [14] and Guatemala [41]. Seyoum et al.*,*[13] reported that almost all the Kenyans have the custom of burning plants to repel mosquitoes. In Guinea Bissau, 55% of people burn plants to repel mosquitoes [40]. The result is also comparable to a study reported by Kweka et al., [34]. The use of plant leaves as insect repellent could be one of more sustainable options than any other parts like roots, resin and bark. This mode of application might not disrupt the natural plant growth as well as it shall supply the leaves throughout the year too [18].

The finding indicates that great majority of the inhabitants apply repellant plants in the early evenings (Figure 3). It could be possibly explained that this happens since the peak biting activity of local malaria vector *An. arabiensis* begins in the dusk hours before people confined with bed nets or other means of interventions [42]. Subsequently residents urge to use repellents in the evenings to evade the insect’s menace and disease transmission. Respondents indicated that these plants are potentially useful, readily accessible and affordable too (Table 3). The findings are in accord with the previous Ethiopian [16-18] and Tanzanian surveys [34], where the majority of the local residents acknowledge that the existing synthetic repellents are not only expensive but also cause dangerous adverse effects than the repellent plants. The studies conducted in Guinea Bissau and Kenya also reported that the majority of the villagers prefer IRPs owing to lack of purchasing power [41,43]. We have personally witnessed and experienced that the Ethiopian IRPs are rationally effective and amiable to apply too.

A chi-square analysis shows a strong association between the respondents’ knowledge on insect repellent plants and the gender (*P* - value = 0.0209), and average monthly income (*P* - value = 0.0226) (Table 4). The findings are quite consistent with the previous Ethiopian studies [16-18]. However, there was no significant association found between the respondents’ knowledge on TIRPs and their age (*P* - value = 0.9979). Result is comparable with the previous studies conducted in Ethiopia with reference to age and knowledge on IRPs [16-18]. It could be possibly explained that since the usage of IRPs is one of the most common practices in the study setting, the residents might have acquired adequate awareness irrespective of their age. Chi-square analysis shows that the repellent plants usage custom is not significantly associated with gender (*P* - value = 0.4420), age (*P* - value = 0.8799), and monthly income (*P* - value = 0.9912) of the respondents (Table 4). It is likely due to the widespread usage of insect repellent plants and long-standing age-old practice and custom. Results are consistent with the earlier studies, which have reported that there is no significant relationship between the age, monthly income of respondents’ and repellent plant usage custom [16-18].

At the moment, there is a revived interest has been observed both among the researchers and general public towards plant-based products attributable to their user-and-eco-friendly nature. It earns more interest as the majority of the commercialized mosquito repellent products are derived from the well-known pyrethrum (Golden Flower) plant [*Asteraceae; Chrysanthemum cinerariaefolium* (current species name: *Tanacetum cinerariifolium*)] from East Africa [44]. Numerous widely-known repellent plants are in use by the indigenous rural people in the SSA countries, though they are quite unaware of the complete elucidation of the mechanism of repellency of those plants [44].

## Conclusion

Arthropods not only serve as disease-transmitters but also cause considerable annoyance in terms of nuisance or menace to the householder. Consequently, in resource-limited settings like Ethiopia people have been applying several repellent plants to repel insects. Ethnobotanical surveys serve as a connecting-link to transfer the practical knowledge and traditional practices from the older to younger generations. It includes basic documentation and quantitative evaluation of the traditional uses of plants as nutraceuticals, and as insect repellents.

The usage of IRPs is a deep-rooted tradition and cultural heritage in Ethiopia. Present survey findings evidently suggest that there is a steady decline/erosion of knowledge and practices of repellent plants. It may be though this survey was conducted in the rural setting, it is located in close proximity of the Jimma town. Therefore, residents might have procured modern personal protection tools than traditional insect repellent plants. Nevertheless, cultural knowledge and traditional practices on insect repellent plants still much more to offer for the humankind. Therefore, it emphasizes on pursuing more ethnobotanical surveys for the proper documentation and preservation of indigenous knowledge and cultural practices. Besides, further studies are required to be warranted to identify and evaluate the responsible bio-active molecules. In addition, measuring their mammalian toxicity is also inevitable [34]. It could lay the first stone to devise affordable user-friendly next generation vector control tools to minimize the vector-borne disease burden especially malaria in the near future.

## Competing interests

The authors declare that they have no competing interests.

## Authors’ contributions

The ethnobotanical survey was initiated by TH and KK. Data was consolidated, interpreted and analyzed retrospectively and independently by KK and TH. KK and TH wrote the manuscript. KK revised the final manuscript. Both authors read and approved the final manuscript.
